# The effect of distance to formal health facility on childhood mortality in rural Tanzania, 2005–2007

**DOI:** 10.3402/gha.v5i0.19099

**Published:** 2012-11-09

**Authors:** Daniel Kadobera, Benn Sartorius, Honorati Masanja, Alexander Mathew, Peter Waiswa

**Affiliations:** 1 Faculty of Health Sciences, University of the Witwatersrand, Johannesburg, South Africa; 2 Makerere University Iganga/Mayuge Health and Demographic Surveillance System, Iganga, Uganda; 3 Ifakara Health and Demographic Surveillance System, Dar es Salaam, Tanzania; 4 Makerere University School of Public Health, Kampala, Uganda; 5 Division of Global Health, IHCAR, Karolinska Institutet, StockholmHagalund, Sweden

**Keywords:** child mortality, Health and Demographic Surveillance System, health facility, distance, Tanzania

## Abstract

**Background:**

Major improvements are required in the coverage and quality of essential childhood interventions to achieve Millennium Development Goal Four (MDG 4). Long distance to health facilities is one of the known barriers to access. We investigated the effect of networked and Euclidean distances from home to formal health facilities on childhood mortality in rural Tanzania between 2005 and 2007.

**Methods:**

A secondary analysis of data from a cohort of 28,823 children younger than age 5 between 2005 and 2007 from Ifakara Health and Demographic Surveillance System was carried out. Both Euclidean and networked distances from the household to the nearest health facility were calculated using geographical information system methods. Cox proportional hazard regression models were used to investigate the effect of distance from home to the nearest health facility on child mortality.

**Results:**

Children who lived in homes with networked distance >5 km experienced approximately 17% increased mortality risk (HR=1.17; 95% CI 1.02–1.38) compared to those who lived <5 km networked distance to the nearest health facility. Death of a mother (HR=5.87; 95% CI 4.11–8.40), death of preceding sibling (HR=1.9; 95% CI 1.37–2.65), and twin birth (HR=2.9; 95% CI 2.27–3.74) were the strongest independent predictors of child mortality.

**Conclusions:**

Physical access to health facilities is a determinant of child mortality in rural Tanzania. Innovations to improve access to health facilities coupled with birth spacing and care at birth are needed to reduce child deaths in rural Tanzania.

The UN Millennium Development Goal Four (MDG 4) aims at reducing child mortality by two-thirds between 1990 and 2015. However, many countries especially in the south Asia and sub-Saharan Africa are not on track to meet this target (1, 2). Child mortality has been declining in the past decade as a result of improved access to new and efficient health services, education, and implementation of child survival programs (2–4), but the decline is not fast enough. The under-five mortality risk in sub-Saharan Africa has decreased from 182 to 142 per 1,000 live births between 1990 and 2008, which is a reduction of about 22% (5) despite the surge in mortality due to the HIV epidemic. A recent analysis of Tanzania Demographic Health Survey (DHS) datasets reported a drop of 24% in child mortality between 2000 and 2004 (6), thus placing Tanzania among the very few African countries that are on course for achieving MDG 4 target on child mortality if the current trend is sustained. Despite this, major improvements are required to cover essential interventions especially for neonates and infants. It has been suggested that most child deaths could be prevented with available and simple low-cost interventions, which are currently not reaching poor children (7). Marginalized groups like these with limited access to health services often stay further away from a needed health infrastructure.

Barriers to access of health facilities still remain important in rural Tanzania. Some authors have characterized access to health in five dimensions: accessibility, availability, affordability, acceptability, and accommodation (8). Geographical accessibility of health facilities by the population has not been adequately assessed especially in terms of distance to health facilities. It has been documented that primary health care usage patterns decline with increasing distance or travel time to a facility. Thus, distance to a health facility is an important factor in determining the utilization of health services in rural areas in low-income countries (9–11). A number of studies have documented the relationship between distance or travel time and health outcomes. Studies in Zambia (12) and Burkina Faso (5, 13) provided evidence that increasing travel time or distance to a health facility was associated with increased child mortality risk. Similarly, in Uganda it was reported that access to a health facility affects childhood mortality and the effect was more evident in children born to uneducated mothers (14).

In Tanzania, infant and child mortality is higher among families living distances more than 5 km away compared to those living <5 km from the nearest health facility. The implications are that even peripheral health facilities still have huge potential to improve the health and survival of families with the current available interventions and resources if distance or travel time can be reduced (15).

On the contrary, transport in rural Tanzania is problematic, be it public or private and often patients have to walk long distances to the nearest health facility, sometimes in difficult terrain (16). In such situations, an assessment of the actual distance travelled can be challenging. Despite the available evidence showing the relationship between access to health facilities and child mortality, some studies have reported the divergent feedback showing that a greater distance to the health care center is not necessarily associated with an increased risk in child mortality (17, 18). This could be attributed to methodological differences and limitations in the measurement of distances in the designs of the studies.

In this study, we used two methods to measure distance travelled to the nearest health facility and its relationship with under-five mortality in rural Tanzania using data from a demographic surveillance site for the period 2005–2007. Euclidean distance is the straight-line distance between two points (e.g. household and health facility), while networked distance is the physical travel path or road a caregiver of a sick child would follow to reach the nearest health facility.

## Materials and methods

### Study area and population

The data for this study was taken from Ifakara Health and Demographic Surveillance System (IHDSS) database for the period 2005–2007. IHDSS started in September 1996 and is found in Ifakara, a rural district in Tanzania characterized by food insecurity, poverty (19), and weak health systems. It is about 320 km from Tanzania's administrative city of Dar es Salaam in the southwestern region of Morogoro and includes 25 villages of Kilombero and Ulanga districts. The area covers 80 km×18 km in Kilombero district and 4 km×25 km in Ulanga district and covers a combined total area of 2,400 km^2^. The two districts are separated by the Kilombero river and the Udzungwa mountains lie to the northwest. The rainy season starts from November to May and annual rainfall ranges between 1,200–1,800 mm with an annual mean temperature of 26°C (20).

The demographic surveillance area (DSA) population has been gradually increasing with 84,000 people living in 19,000 scattered rural households at the time of analysis. The population is ethnically heterogeneous although the national language is Swahili with a population density of 35 people/km^2^. The majority of the population practice Christianity (60%) or Islam (40%) (20).

The population is predominantly rural, practicing subsistence farming and fishing as the main occupations with rice and maize – the predominant food crops. Most families live in mud walls and grass-thatched roof houses, but they also have a second house known as shamba house (farmhouse), where they stay during the planting and harvesting seasons. The population is highly mobile with most families moving between the main home and the shamba home, depending on the season. Shallow/open wells and rivers are the common sources of water in the area, and the majority of the homes do not have electricity. Infectious diseases are the main causes of mortality and morbidity with malaria causing the biggest burden (20). Since January 1997, all deaths, births, pregnancies, and migrations are recorded during update household visits conducted every 4 months. These vital events are collected for each household as well as new households that have emerged since the last visit. During the update round, a trained field worker interviews an adult or the most knowledgeable respondent aged 18 and above by cross checking all individual information in the register recorded at the last visit and collects all the events that occurred since the previous update round. The data quality was considered good as it was assessed using basic demographic indicators such as sex ratios at births and population structure.

The public health system in the DSA consists of village health workers, dispensaries, health centers, and hospitals. There are a total of 13 dispensaries and 2 health centers in the DSA providing preventive and curative services to the population. In addition, there are two district hospitals outside the DSA. The immunization rates are above 80% and malaria is the leading cause of admission and OPD for both children and adults in the area (20).

### Study design, data collection, management, and analysis

This was a retrospective population-based cohort study using a longitudinal dataset collected by the IHDSS. Secondary data analysis was carried out on the data collected on children younger than age five residing in the DSA. The study included all eligible children younger than age 5 between 2005 and 2007 living in households with coordinates. About 18% of the households in IHDSS were excluded in the analysis because of missing household coordinates. Children were censored on 31 December 2007 if still alive and <5 years, at age 5 if this occurred within the study period, or if they out-migrated within this period before age 5.

All persons who were 5 years and above on 1 January 2005 and resident in the Ifakara Health DSA were excluded from the study.

Our main explanatory variable was network distance. This is a more complex version of distance estimation that necessitates path and road network files and could include directions, speed limits, barriers, type of road surface to produce a more accurate measurement. Network distance greatly minimizes errors attributed to subjective reporting of distances or travel time by the respondents. Road surface, barriers and speed limits could not be collected in this study and are therefore missing in the computation of the network distance variable.

The study outcome was child deaths that occurred between January 2005 and December 2007, which were captured by field interviewers during quarterly follow-up visits to the households.

Data management and analysis was done in STATA version 10 (21). Missing values, inconsistent and incomplete data were all identified and corrected in STATA during the cleaning. Geographical information systems (GIS) methods were used to analyze the main exposure variable, that is, distance. Network distance and Euclidean (straight-line) distance from home to the nearest health facility were the two types of distance that were analyzed in this study. The Ifakara HDSS had all households, roads and small paths, health facilities, and schools in the DSA georeferenced in 2006. With the help of ArcView 9.1 software, three shape files were generated, that is, roads/small paths shape file, household shape file, and health facility shape file. Using the network analyst tool in ArcView, a routes feature layer was generated after the analysis, which stored the resultant shortest networked and Euclidean routes from households to the closest health facility.

Data were analyzed using the Cox proportional hazard regression models. Kaplan–Meier survival curves were estimated for different subgroups of network distance and compared with log rank test. The network and Euclidean distance, which were the main explanatory variables, were entered in the cox regression to determine the association between distance to the nearest health facility and infant and child mortality while adjusting for confounding factors like age of child, gender, mother's age, mother's education, mother's age, death of mother, death of preceding sibling, and district of residence.

The Ifakara Health Institute (IHI) Institutional Review Board (IRB) and Wits Human Research Ethics Committee (Approval number R14/49) provided ethical approval.

## Results

### Socio demographic characteristics

A total of 23,823 children younger than age 5 living in 13,845 households registered in the IHDSS between 2005 and 2007 were included in the analysis. Among these, 917 under-five deaths were recorded in both districts. The sociodemographic characteristics of children in the study are shown in Table 1. There were 748 (81.6%) infant deaths and 169 (18.4%) child (1–4) deaths in the study. A total of 4.2% of deaths occurred among male children while 3.5% died among the female children. About 778 (4.8%) deaths occurred among children living with their mothers while 81 (12.7%) deaths occurred among children borne of a multiple birth outcome. There were 39 (14.4%) child deaths reported among children whose previous sibling had died. The mean birth interval to previous sibling was 36.6 months while the next sibling was 37.1 months. The mean straight-line distance was 4.2 km (SD 3.9 km) while the mean network distance from home to nearest health facility was 8.3 km (SD 6.8 km) within the study area. Farming was the predominant occupation of household heads in the study. About 18% of the households in IHDSS were excluded because of missing georeference codes. The excluded households were compared to the included households in terms of various sociodemographic characteristics and we found that the missing georeferences were non-random.

**Table 1:** Socio-demographic characteristics of the children in the IDHSS between 2005 and 2007

Variable	Dead: *n* (%)	Alive: *n* (%)	Total: *n* (%)
District (*n*=23,823)			
Kilombero	556 (3.9)	13,657 (96.1)	14,213 (100)
Ulanga	361 (3.8)	9,249 (96.2)	9,610 (100)
Gender (*n*=23,823)			
Male	502 (4.2)	11,366 (95.8)	11,955 (100)
Female	415 (3.5)	11,540 (96.5)	11,868 (100)
Age (Mean, SD)	1.6	1.7	23,823
Family size^1^ (*n*=23,823)			
1–2	505 (3.5)	14,107 (96.5)	14,612 (100)
3+	412 (4.5)	8,799 (95.5)	9,211 (100)
Season (*n*=23,823)			
Rainy	285 (3.9)	6,971 (96.1)	7,256 (100)
Dry	632 (3.8)	15,935 (96.2)	16,567 (100)
Co-residence of mother (*n*=23,823)			
Yes	778 (4.8)	15,320 (95.2)	16,098 (100)
No	139 (1.8)	7,586 (98.2)	7,725 (100)
Mothers age^1^ (*n*=16,098)			
< 20	180 (7.0)	2,399 (93.0)	2,579 (100)
20–29	354 (4.6)	7,425 (95.5)	7,779 (100)
30–30	219 (4.6)	4,513 (95.4)	4,732 (100)
40+	25 (2.5)	983 (97.5)	1,008 (100)
Multiple birth^1^ (*n*=16,098)			
Multiple	81 (12.7)	555 (87.3)	636 (100)
Single	697 (4.5)	14,765 (95.5)	15,462 (100)
Mother death^1^ (*n*=16,098)			
Yes	37 (19.0)	158 (81.0)	195 (100)
No	741 (4.7)	15,162 (95.3)	15,903 (100)
Mothers education^1^ (*n*=12,172)			
None	87 (4.7)	1,781 (95.3)	1,868 (100)
Primary	430 (4.3)	9,685 (95.8)	10,115 (100)
Post primary	5 (2.7)	184 (97.4)	189 (100)
Parity^1^ (*n*=11,774)			
1	163 (7.9)	1,889 (92.1)	2,052 (100)
2–3	245 (5.4)	4,289 (94.6)	4,534 (100)
4+	303 (5.8)	4,885 (94.2)	5,188 (100)
Wealth quintiles^1^ (*n*=23,302)			
Poorest	158 (4.2)	3,572 (95.8)	3,730 (100)
Poorer	164 (3.7)	4,333 (96.4)	4,497 (100)
Poor	203 (3.8)	5,165 (96.2)	5,368 (100)
Less poor	183 (3.9)	4,495 (96.1)	4,678 (100)
Least poor	186 (3.7)	4,843 (96.3)	5,029 (100)
HHH* occupation^1^ (*n*=23,154)			
Farming	633 (3.9)	15,587 (96.1)	16,220 (100)
Causal worker	36 (3.4)	1,036 (96.6)	1,072 (100)
Business	107 (3.8)	2,712 (96.2)	2,819 (100)
Gov't employee	80 (4.1)	1,859 (95.9)	1,939 (100)
Other	34 (3.1)	1,070 (96.9)	1,104 (100)
Death of previous sibling (*n*=23,823)			
No	878 (3.7)	2,265 (96.3)	23,553 (100)
Yes	39 (14.4)	231 (85.6)	270 (100)
Birth interval to next sibling (months) (Mean, SD)	37.1	13.9	4,737
Birth interval to previous sibling (months) (Mean, SD)	36.6	14.2	5,217

* Household head.

1 Variable has some missing values.

### Mortality

**Fig. 1:**
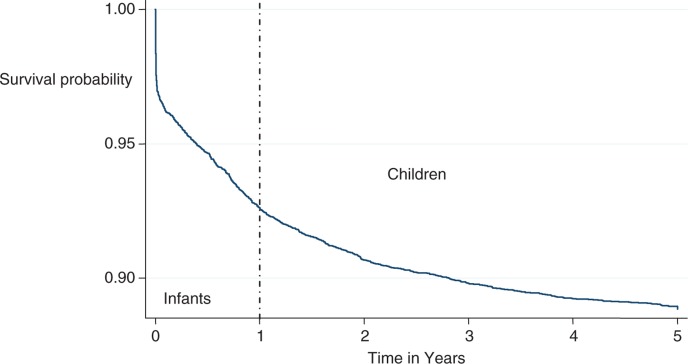
Kaplan–Meier graph showing overall survival of under-five children in Ifakara HDSS.

A total of 23,823 children were assessed and these contributed a total of 37456.64 person years of observation over a period of 3 years. Figure 1 shows the survival probability for infants, children, and under-five children for the period 2005–2007. The results show very high mortality during infancy compared to the childhood.

### Infant and child mortality rates by networked distance

Both the infant and child mortality rates increased as network distance from a health facility increased. The mortality rate for infants who lived <5 km network distance to the nearest health facility was 72.4 (95% CI 63.5–82.5) per 1,000 person years while the mortality rate for those living >5 km from the health facility was 82.3 (95% CI 74.6–90.7) per 1,000 person years. The child mortality rates also followed the same trend, increasing as network distance to the nearest health facility increased with 8.4 (95% CI 6.2–10.9) and 9.9 (95% CI 8.2–12.0) deaths per 1,000 person years of observation for <5 km and ≥5 km categories, respectively.

### Child survival in IHDSS between 2005–2007

The survival probability of under-five children improved as age increased (Fig. 1). The survival probability dropped sharply during the initial infancy when compared to the child (1–4) stage. The figure shows that the risk of mortality is most severe during the infant stages of childhood especially during the neonatal stage as depicted by the sharp fall of the graph at the beginning. The survival hazard at 1 year was 0.93 (95% CI 0.920–0.932) and 0.89 (95% CI 0.88–0.90) at 5 years. About 8% of the children died in their first year of life but survival of the children in the study thereafter improved as they entered the child (1–4) stage with 5% deaths recorded in this stage before their fifth birthday.

**Fig. 2:**
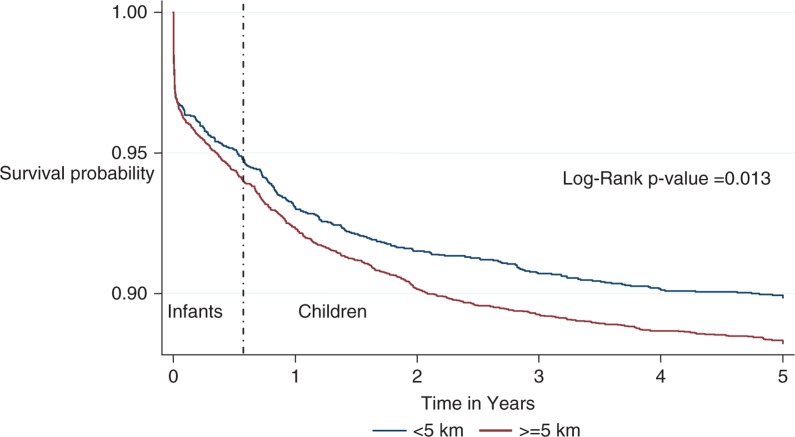
Kaplan–Meier survival graphs by networked distance of under-five children in Ifakara HDSS.

The survival probability appeared higher at all-time points (Fig. 2) throughout the childhood stage for children who lived <5 km network distance compared to those who lived >5 km network distance. The difference between the survival probability of the children who lived <5 km and >5 km network distances to the health facility seemed to be bigger during the child (1–4) stage when compared to the infant stage (*p*-value for log rank test=0.013). This shows that as the children grew older, those who lived >5 km networked distance had a much higher decline in their survival probability when compared to those who lived <5 km away.

In the Cox regression analysis, network distance to the nearest health facility was strongly associated with under-five mortality after controlling for gender, maternal age, mother death, multiple birth, parity, death of preceding sibling, and family size. Following multivariate analysis, residing more than 5 km network distance to the nearest health facility was associated with 1.17 (95% CI: 1.02, 1.38) increased child mortality hazard compared to those children who lived <5 km network distance to the nearest health facility. Infant (<1 year) and child (1–4 years) models posted insignificant results with network distance at the multivariate level. Death of a mother (HR=5.87; 95% CI 4.11–8.40), death of preceding sibling (HR=1.9; 95% CI 1.37–2.65), and multiple births (HR=2.9; 95% CI 2.27–3.74) were the strongest independent predictors of child mortality (Table 2).

**Table 2:** Univariate and multivariate analysis of under-five mortality

	Univariate		Multivariate	
		
Variable	HR	90% CI	HR	95% CI
District				
Kilombero (Ref)				
Ulanga	0.97	0.86–1.10		
Gender				
Female (Ref)				
Male	1.22	1.09–1.37	1.16	1.01–1.35
Age at start of study	0.49	0.45–0.52	0.35	0.29–0.43
Euclidean distance				
< 5 km (Ref)				
> 5 km	1.08	0.96–1.21		
Networked distance				
< 5 km (Ref)				
> 5 km	1.19	1.06–1.33	1.17	1.02–1.38
Mothers age				
< 20 (Ref)				
20–29	0.59	0.51–0.69	0.93	0.74–1.17
30–39	0.59	0.50–0.69	1.21	0.89–1.64
> 40	0.32	0.22–0.45	1.00	0.611–1.64
Mother education				
None (Ref)				
Primary	0.91	0.75–1.10		
Post primary	0.56	0.26–1.19		
Parity				
1 (Ref)				
2 to 3	0.65	0.55–0.77	0.61	0.52–0.83
4+	0.69	0.59–0.81	0.56	0.52–0.91
Multiple/single birth				
No(Ref)				
Yes	2.99	2.46–3.63	2.91	2.27–3.74
Mother death				
No(Ref)				
Yes	4.41	3.34–5.82	5.87	4.11–8.40
Birth interval to previous sibling	1.00	0.99–1.00		
Birth interval to next sibling*	0.91	0.90–0.92		
Death of preceding sibling				
No(Ref)				
Yes	4.5	3.42–5.91	1.90	1.37–2.65
Season				
Rainy(Ref)				
Dry	0.95	0.85–1.07		
Wealth quintiles**				
Poorest (Ref)				
Poorer	0.86	0.71–1.02		
Poor	0.88	0.73–1.04		
Less poor	0.91	0.76–1.09		
Least poor	0.86	0.72–1.02		
HHH occupation				
Farming (Ref)				
Causal worker	0.85	0.64–1.12		
Business	0.95	0.80–1.12		
Gov't employee	1.09	0.90–1.33		
Other	0.79	0.59–1.05		
Family size				
1–2(Ref)				
3+	1.36	1.22–1.52	1.30	1.11–1.52

* Not included in multivariate because of small numbers.

** Wealth Index showed significant association with child (1–4) mortality though no association existed with infant mortality in the analysis.

## Discussion

This study explored the association between distance to the nearest health facility and under-five mortality in IHDSS in rural Tanzania while adjusting for other important determinants. Our findings provide further evidence on the effect of increasing under-five children mortality risk as network distance from home to the nearest health facility increases. The under-five mortality risk clearly increases as distance increases. These findings are similar to earlier studies (5, 12, 14, 15, 22, 23) that found an effect of distance to the nearest health facility on child mortality.

We found that network distance to the nearest health facility was a better estimator of distance than the Euclidean distance. Both measurements appeared to have a more consistent effect on child mortality rates than infant mortality rates. This could be attributed to the fact that Euclidean distances are hypothesized travel distances to the nearest health facility unlike the network distance measures, which tend to measure the more actual travel distances to the health facility. They demonstrate actual paths or roads that an ill child would probably follow to the nearest health facility which are much longer and more realistic. Although Euclidean and network distances are strongly correlated, variations still exist especially in suburban and rural areas (24). It is recommended that in measuring distances in rural areas, network based distance may provide the best estimates for the purpose of health research (25). In addition, we know that most infants die during the neonatal period mainly because of issues related to the quality of care at the time of birth (26, 27).

The network distance to the nearest health facility provided the study with the best estimation for geographical accessibility under the circumstances since there was no reliable data on transport means, seasonality of the roads, speeds limits and geographical barriers like rivers and lakes which could be explored in future studies. Inclusion of these factors in the estimation of the network distance greatly improves the accuracy of the network estimation and makes it more realistic. Thus, evaluations involving distance as a measure of access should employ the networked distance, as it is a better measure.

Many studies (22, 28–31) have documented the major risk factors for child mortality especially in sub Saharan Africa. Similarly, gender, multiple birth, maternal death, death of preceding sibling, and bigger family size were found to be significantly associated with increased child mortality risk while age of child, higher maternal age, higher parity, and birth interval to next sibling were also shown to have a protective effective on the under-five mortality in the study.

Our finding of a strong association of a mother's death during childhood by the study was also found in studies in SSA (13, 22), which underlines the big role mothers have to play in the survival of children. For instance, it is estimated that for every maternal death attributed to HIV, there are two orphaned children and with the current HIV epidemic devastating SSA, loss of the primary caregiver like a mother could possibly explain the high childhood mortality in these areas (32). A multiple birth in the study experienced 2.91 times greater mortality compared to single births, similar to findings from other studies (13, 33). A study in southern and eastern Africa (33) demonstrated the magnitude of excess mortality for twin births over single births, although the excess mortality was largest during the first year of life. Mothers are usually stretched by the extra responsibility multiple births demand, with some mothers suffering from insufficient breast milk which reduces the immunity of the babies in the early stages of life leading to the high child mortality incidence.

### Methodological considerations

This study had several limitations. Distances are likely to contain measurement error due to incorrect geocoordinates and in the case of network distance, difficulties in terrain and seasonality were not considered in the measurement. The study used available data in the IHDSS for analysis and was therefore limited to variables that were present in the dataset. Inclusion of data on factors like marital status, religion, and health facility-based variables like drug and vaccine stock, number of staff, and availability of water could have heavily enriched the results of the study. Further, reliability and validity of the study results are dependent on how well the data were collected by the IHDSS field staff. However, the HDSS platform provides rigorous repeated visits of households at regular intervals, which ensures consistency and accuracy of the data.

In demographic surveillance, there is free entry and exit of households in the study area. Therefore, households that dissolved in 2005 before the start of georeferencing of all households in 2006 in the DSA could introduce selection bias in case the dissolutions were not random.

## Conclusions

Distance to health facilities is a key predictor of child mortality in rural Tanzania. Innovative strategies, such as outreaches, improved transportation, use of community health workers, and strengthening of first-level health units, are needed to decrease physical barriers to access health services by the marginalized populations. Future studies assessing access to health care should integrate network distance and quality of care at the facilities to show how the two variables inform the decision of choice of care.

## References

[1] Black RE, Cousens S, Johnson HL, Lawn JE, Rudan I, Bassani DG (2010). Global, regional, and national causes of child mortality in 2008: a systematic analysis. Lancet.

[2] You D, Wardlaw T, Salama P, Jones G (2010). Levels and trends in under-5 mortality, 1990–2008. Lancet.

[3] Pison G, Trape JF, Lefebvre M, Enel C (1993). Rapid decline in child mortality in a rural area of Senegal. Int J Epidemiol.

[4] Delaunay V, Etard J-F, Préziosi M-P, Marra A, Simondon F (2001). Decline of infant and child mortality rates in rural Senegal over a 37-year period (1963–1999). Int J Epidemiol.

[5] Schoeps A, Gabrysch S, Niamba L, Sié A, Becher H (2011). The effect of distance to health-care facilities on childhood mortality in rural Burkina Faso. Am J Epidemiol.

[6] Masanja H, de Savigny D, Smithson P, Schellenberg J, John T, Mbuya C (2008). Child survival gains in Tanzania: analysis of data from demographic and health surveys. Lancet.

[7] Bryce J, Victora C, Habicht J, Black R, Scherpbier R, Advisors tM-IT (2005). Programmatic pathways to child survival: results of a multi-country evaluation of Integrated Management of Childhood Illness. Health Policy Plan.

[8] Penchansky R, Thomas JW (1981). The concept of access: definition and relationship to consumer satisfaction. Med Care.

[9] Muller I, Smith T, Mellor S, Rare L, Genton B (1998). The effect of distance from home on attendance at a small rural health centre in Papua New Guinea. Int J Epidemiol.

[10] Tanser F, Gijsbertsen B, Herbst K (2006). Modelling and understanding primary health care accessibility and utilization in rural South Africa: an exploration using a geographical information system. Soc Sci Med.

[11] Thaddeus S, Maine D (1994). Too far to walk – maternal mortality in context. Soc Sci Med.

[12] Kapungwe A (2005). Quality of child health care and under-five-mortality in Zambia: a case study of two districts in Luapula Province. Demogr Res.

[13] Becher H, Muller O, Jahn A, Gbangou A, Kynast-Wolf G, Kouyate B (2004). Risk factors of infant and child mortality in rural Burkina Faso. Bull World Health Organ.

[14] Katende C (2003). The impact of access to health services on infant and child mortality in rural Uganda. Popultion Studies.

[15] Armstrong Schellenberg J, Mrisho M, Manzi F, Shirima K, Mbuya C, Mushi A (2008). Health and survival of young children in southern Tanzania. BMC Public Health.

[16] Masuma M, Bangser M (2009). Poor people's experiences of health services in Tanzania.

[17] Dummer T, Parker L (2004). Hospital accessibility and infant death risk. Arch Dis Child.

[18] Goodman DC, Fisher E, Stukel TA, Chang C (1997). The distance to community medical care and the likelihood of hospitalization: is closer always better?. Am J Public Health.

[19] Marchant T, Schellenberg JA, Nathan R, Abdulla S, Mukasa O, Mshinda H (2004). Anaemia in pregnancy and infant mortality in Tanzania. Trop Med Int Health.

[20] Schellenberg A, Oscar M, Salim A, Tanya M, Christian L, Nassor K (2002). Ifakara DSS, Tanzania. Population and health in developing countries.

[21] StataCorp (2007). Statistical Software Release. 10.0 In: Release 10 ED ed.

[22] Hammer GP, Kouyaté B, Ramroth H, Becher H (2006). Risk factors for childhood mortality in sub-Saharan Africa: a comparison of data from a demographic and health survey and from a demographic surveillance system. Acta Trop.

[23] Frankenberg E (1992). Infant and early childhood mortality in Indonesia: the impact of access to health facilities and other community characteristics on mortality risks.

[24] Apparicio P, Abdelmajid M, Riva M, Shearmur R (2008). Comparing alternative approaches to measuring the geographical accessibility of urban health services: distance types and aggregation-error issues. Int J Health Geogr.

[25] Brabyn L, Skelly C (2002). Modeling population access to New Zealand public hospitals. Int J Health Geogr.

[26] Lawn JE, Cousens S, Darmstadt GL, Paul V, Martines J (2004). Why are 4 million newborn babies dying every year?. Lancet.

[27] Lawn JE, Cousens S, Zupan J (2005). 4 million neonatal deaths: when? Where? Why?. Lancet.

[28] Mturi AJ, Curtis SL (1995). The determinants of infant and child mortality in Tanzania. Health Policy Plan.

[29] Binka FN, Maude GH, Gyapong M, Ross DA, Smith PG (1995). Risk factors for child mortality in northern Ghana: a case-control study. Int J Epidemiol.

[30] Gemperli A, Vounatsou P, Kleinschmidt I, Bagayoko M, Lengeler C, Smith T (2004). Spatial patterns of infant mortality in Mali: the effect of malaria endemicity. Am J Epidemiol.

[31] Vella V, Tomkins A, Nidku J, Marshall T (1992). Determinants of child mortality in South-West Uganda. J Biosoc Sci.

[32] Quinn TC (1996). Global burden of the HIV pandemic. Lancet.

[33] Justesen A, Kunst A (2000). Postneonatal and child mortality among twins in Southern and Eastern Africa. Int J Epidemiol.

